# Spatial Distribution and Fuzzy Health Risk Assessment of Trace Elements in Surface Water from Honghu Lake

**DOI:** 10.3390/ijerph14091011

**Published:** 2017-09-04

**Authors:** Fei Li, Zhenzhen Qiu, Jingdong Zhang, Chaoyang Liu, Ying Cai, Minsi Xiao

**Affiliations:** 1 Research Center for Environment and Health, Zhongnan University of Economics and Law, Wuhan 430073, China; lifei@zuel.edu.cn (F.L.); zzqiu@zuel.edu.cn (Z.Q.); lcy@zuel.edu.cn (C.L.); 1993cy@zuel.edu.cn (Y.C.); msxiao@zuel.edu.cn (M.X.); 2 School of Information and Safety Engineering, Zhongnan University of Economics and Law, Wuhan 430073, China

**Keywords:** surface water, trace elements, spatial distribution, health risk assessment, triangular fuzzy numbers, Honghu Lake

## Abstract

Previous studies revealed that Honghu Lake was polluted by trace elements due to anthropogenic activities. This study investigated the spatial distribution of trace elements in Honghu Lake, and identified the major pollutants and control areas based on the fuzzy health risk assessment at screening level. The mean total content of trace elements in surface water decreased in the order of Zn (18.04 μg/L) > Pb (3.42 μg/L) > Cu (3.09 μg/L) > Cr (1.63 μg/L) > As (0.99 μg/L) > Cd (0.14 μg/L), within limits of Drinking Water Guidelines. The results of fuzzy health risk assessment indicated that there was no obvious non-carcinogenic risk to human health, while carcinogenic risk was observed in descending order of As > Cr > Cd > Pb. As was regarded to have the highest carcinogenic risk among selected trace elements because it generally accounted for 64% of integrated carcinogenic risk. Potential carcinogenic risk of trace elements in each sampling site was approximately at medium risk level (10^−5^ to 10^−4^). The areas in the south (S4, S13, and S16) and northeast (S8, S18, and S19) of Honghu Lake were regarded as the risk priority control areas. However, the corresponding maximum memberships of integrated carcinogenic risk in S1, S3, S10–S13, S15, and S18 were of relatively low credibility (50–60%), and may mislead the decision-makers in identifying the risk priority areas. Results of fuzzy assessment presented the subordinate grade and corresponding reliability of risk, and provided more full-scale results for decision-makers, which made up for the deficiency of certainty assessment to a certain extent.

## 1. Introduction

Surface waters are important sources of domestic water for humans, but they are more sensitive and vulnerable to contaminants due to the extensive area and accessibility to wastewater. Surface waters have been polluted owing to wide acceptance of domestic sewage, industry wastewater and agricultural wastewater in China. Contaminations, including organic pollutants, trace elements and elevated nutrients are the origins of the pollution. Moreover, trace elements have been confirmed to have potential biological risk, ecological risk, and health risk [1,2,3,4]. The pollution of trace elements in surface water has drawn particular attention globally, and has been widely studied with respect to migration mechanisms [5], toxicity [6], accumulation mechanism [7], ecological hazards [8] and the impact on human health [9,10].

Honghu Lake is a national nature reserve in Hubei Province, and was listed in the Ramsar Convention in 2008. It plays a key role in domestic activities, water storage, irrigation, fisheries and shipping. Unfortunately, with rapid growth of agriculture and industry, Honghu Lake suffered from a certain degree of pollution. It has been concerned more with pollution effects on biodiversity [11] and the ecological environment [12]. Previous studies of the aquatic environment in Honghu Lake were mainly focused on water eutrophication, distribution characteristics of trace elements, and ecosystem destruction of water pollution. Studies revealed that Honghu Lake was polluted by trace elements due to anthropogenic activities [12,13]. There are a few studies on potential hazards of trace elements to human health at present. Furthermore, some farmers and fishermen take preliminarily preprocessed water from Honghu Lake for domestic water and drinking water. Therefore, it is essential to evaluate potential human health risk exposure by trace elements in surface water from Honghu Lake and to identify the priority pollutants and control areas.

At present, the health risk assessment system issued by United States Environmental Protection Agency (USEPA) has an increasingly wide utilization in many countries and achieved good results. However, the certainty health risk assessment method has some parameter uncertainties and needs to be improved, mainly embodied in the following points: (1) The limitation of the pollutants’ heterogeneity in temporal and spatial distribution leads to the fuzziness of pollutant concentrations; (2) with limited manpower and material resources, the sampling sites are often limited to a certain extent; (3) there are individual and regional differences in human exposure characteristics to pollutants. Gender, age, season, occupation and other factors could lead to personal uncertainty; (4) the selected acceptable risk levels vary among different countries, cities and regions, which may lead to largely different risk assessment conclusions and management policies; (5) there are unavoidable random errors in the detection of pollutant contents, which results in the randomness of pollutant concentrations. All the above parameter uncertainties may eventually lead to an unexpected volatility of health risk results. Therefore, triangular fuzzy numbers were introduced into the health risk assessment in order to reduce the parameter uncertainty and provide more objective and comprehensive results for decision-makers.

The objectives of this study were (1) to investigate the total content and spatial distribution of trace elements in surface water from Honghu Lake; (2) to carry out the health risk assessment at a screening level for the trace elements on receptors based on triangular fuzzy numbers; (3) to further study the carcinogenic risk levels and corresponding reliability degrees in order to identify the major pollutants and control areas, and judge whether Honghu Lake, as a drinking water source, threatens human health.

## 2. Materials and Methods

### 2.1. Study Area

Honghu Lake, the seventh largest freshwater lake in China, is also the largest natural lake in Hubei Province. Honghu Lake is located in Southern Jianghan Plain at the end of the Four-lake Main Canal (Chang Lake, San Lake, Bailu Lake and Honghu Lake), and spans Honghu City and Jianli City. Honghu Lake is an obstruction lake in the depression between the Yangtze River and Dongjing River, with abundant aquatic animals and plant resources. It is in a shape of polygon with a catchment area of 355 km². The average water depth of Honghu Lake is only 1.35 m, with a maximum depth of 2.32 m in the wet season, so it is a typical large, shallow, weedy lake. Honghu Lake is a semi-closed lake, and the inflows are mainly from the north through the Four-lake Main Canal. Annual average water volume flowing into Honghu Lake is 1.96 × 10^9^ m^3^. Outflow of Honghu Lake discharges into the Yangtze River through the only gate in the southeast, and the gate is open during the flood period. The characteristic of being semi-closed determines the relatively low fluidity of surface water in Honghu Lake. The fluctuation of the water level in Honghu Lake mainly depends on precipitation of the four lakes and runoff from upper reaches. Honghu is the largest lake in the Four-lake Watershed with the strongest storage capacity. Therefore, it is a representative and typical lake on the Jianghan Plain and even the middle and lower reaches of the Yangtze River.

### 2.2. Samples Collection and Detection

**Figure 1:**
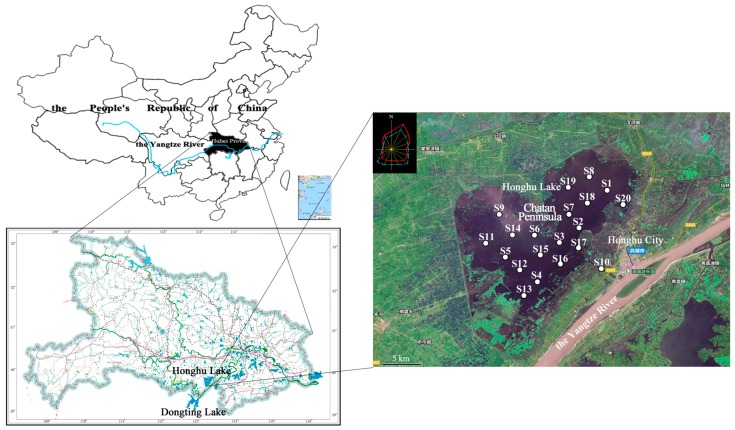
Map of surface water sampling sites in Honghu Lake.

Twenty water samples were collected from Honghu Lake during August, 2016, for a screening-level assessment. The sampling time was confirmed by comprehensive consideration of hydrologic features, climate characteristic and the latest situation in Honghu Lake (details are shown in Supplementary Material). The designed layout of sampling points in the lake strictly referred to “Technical Specifications Requirements for Monitoring of Surface Water and Waste Water (HJ/T 91–2002)” [14] and was evenly distributed with the mesh method based on the lake area and local hydrology situation. According to “Water Quality—Guidance on Sampling Techniques (HJ 494–2009)”, the sampling depth was determined at one-quarter of the water depth below surface [15]. The sampling sites were arranged with geographic coordinates (latitude and longitude) in advance. A hand-held Global Positioning System (GPS) was applied for navigation of the scene. Sampling sites are shown in Figure 1, and hereafter referred to as S1–S20.

All water samples were filtered through 0.45 μm millipore filters, and then were collected into 1 L pre-conditioned acid-washed polyethylene containers [16]. The sampling containers were rinsed at least three times with the fresh water from Honghu Lake before collecting the water samples. Subsequently, water samples were acidified to pH 1–2 with HNO_3_ (GR), and then they were stored in thermostats with ice for transport to laboratory. In addition, parallel and blank samples were also collected, and underwent the same operations as the water samples. Physicochemical parameters including pH, temperature, dissolved oxygen (DO) and electrical conductivity (EC) were measured by a multi-parameter water quality analyzer (HD40Q, HACH, Loveland, CO, USA) in the field.

Digestion methods of water samples were referred to “Water Quality–Digestion of Total Metals-Nitric Acid Digestion Method (HJ 677–2013)” [17] and “Water Quality—Determination of Mercury, Arsenic, Selenium, Bismuth and Antimony–Atomic Fluorescence Spectrometry (HJ 694-2014)” [18]. After digestion, the total amount of Cu, Zn, Cr, Cd and Pb was detected with Atomic Absorption Spectroscopy (AAS ZEEnit 700P, Jena, Germany) and As was detected by Atomic Fluorescence Spectrometry (AFS-9730, Haiguang Instrument Co. Ltd., Beijing, China) under appropriate analytical conditions. Quality assurance and quality control were carried out with parallel experiments, blank tests and recovery tests. The recovery rates were between 90% and 110%, and relative deviations of parallel tests were within 10%.

### 2.3. Fuzzy Health Risk Assessment of Trace Element

#### 2.3.1. Health Risk Assessment Model

Health risk assessment is identified as the processes to estimate events probability and probable degree of adverse health effects over a specific period [19]. Risk level of environmental pollutants to human beings depends on the body’s exposure dose to the pollutants and toxicity of pollutants. There are two main pathways for human exposure to trace elements in water: ingestion and dermal absorption, ignoring the exposure via inhalation [3,20,21]. The exposure dose can be calculated by Equations (1) and (2) [22,23].

(1) $${\mathrm{ADD}}_{\mathrm{ing}}=\frac{C_{w}\times \mathrm{IR}\times \mathrm{EF}\times \mathrm{ED}}{\mathrm{BW}\times \mathrm{AT}}$$

where ADD_ing_ (μg/(kg·day)) represents the exposure dose through ingestion, in this study, the ingestion mainly refers to the intake through water from Honghu Lake; C_w_ is the mean concentration of trace element in water (μg/L); IR is the intake rate of water, including direct drinking rate and indirect drinking rate (L/day); EF is the exposure frequency to pollutants (day/year); ED is the exposure duration, and it means the length of time over which contact with the contaminant lasts (year); BW represents the body weight (kg); AT is the average time (day). For carcinogenic risk, AT is the average life expectancy of people; for non-carcinogenic risk, AT is equal to ED×365 [23].

(2) $${\mathrm{ADD}}_{\mathrm{derm}}=\frac{C_{w}{\times \mathrm{SA}\times K}_{p}{\times \mathrm{ET}\times \mathrm{EF}\times \mathrm{ED}\times 10}^{-3}}{\mathrm{BW}\times \mathrm{AT}}$$

where ADD_derm_ (μg/(kg·day)) represents the exposure dose through dermal absorption; SA is the exposure area of skin (cm^2^); K_p_ is the dermal permeability coefficient of pollutants in water (cm/h), in this study, 0.001 cm/h for Cu, Cd and As, 0.0001 cm/h for Pb, 0.002 cm/h for Cr, and 0.0006 cm/h for Zn [3,20]; and ET is the exposure time (h/day), in this study, ET is 0.6 h/day [3]. For the meanings of C_w_, EF, ED, BW, and AT, please refer to Equation (1).

The health risks caused by environmental pollutants can be divided into carcinogenic risk and non-carcinogenic risks according to the properties. Non-carcinogenic risk takes hazard quotient (HQ) as the measure of risk assessment. As shown in Equation (3) [23], HQ is the ratio of daily exposure dose to the reference dose. If synergy and antagonism between different pollutants are not considered, integrated non-carcinogenic risk which represented by hazard index (HI) is the sum of HQs caused by various pollutants through different pathways [24]. HI > 1 means a certain degree of adverse effects on human health; HI ≤ 1 indicates no harm [23,25].

(3) $${\mathrm{HQ}}_{i}=\frac{{\mathrm{ADD}}_{i}}{{\mathrm{RfD}}_{i}}$$

(4) $$\mathrm{HI}=\overset{n}{\underset{i=1}{\sum }}{\mathrm{HQ}}_{i}$$

where HQ*_i_* is the hazard quotient of trace elements through ingestion or dermal absorption, dimensionless; ADD*_i_* (μg/(kg·day)) is the daily exposure dose of non-carcinogenic pollutants; RfD (μg/(kg·day)) is the reference dose of pollutants; *i* is the pathways of exposure; *n* is the kinds of trace elements; HI is the hazard index, which is the sum of HQs of the studied trace elements from all the applicable pathways, in this study, and the pathways include ingestion and dermal absorption.

Carcinogenic risk is the product of daily exposure dose and cancer slope factor, which is shown in Equation (5). Under the assumption that there is no antagonism and synergism between pollutants, the integrated carcinogenic risk can also be identified as the sum of carcinogenic risks exposure by various pollutants via different pathways. USEPA believes that carcinogenic risk value of human being is acceptable within 1 × 10^−4^ [26], while the maximum acceptable risk value recommended by International Commission on Radiological Protection (ICRP) is 5 × 10^−5^ [27]. The significant difference between the two evaluation standards may mislead the decision makers in their final judgment. Furthermore, it should be noted that there is currently no official and uniform standard of acceptable risk value in China and many developing countries, which may lead to uncertainty and incomparability among different decision-makers. Therefore, risk classification was carried out in this study in order to make the evaluation results clearer and more intelligible. Risk levels were rated as 7 levels based on the Delphi method, assessment criteria of USEPA and ICRP, as well as existing research (Table 1) [28,29,30,31].

(5) $${\mathrm{CR}}_{i}{=\mathrm{ADD}}_{i}{\times \mathrm{CSF}}_{i}$$

(6) $$\mathrm{CR}=\overset{n}{\underset{i=1}{\sum }}{\mathrm{CR}}_{i}$$

where CR*_i_* is the carcinogenic risk of trace elements through ingestion or dermal absorption, dimensionless; ADD*_i_* (μg/(kg·day)) is the daily exposure dose of carcinogenic pollutants; CSF*_i_* (kg·day/μg) is the cancer slope factor of carcinogenic pollutants; CR is the sum of CR*_i_*; for *n* and *i*, please refer to Equation (4).

#### 2.3.2. Exposure Parameters Selection Based on Triangular Fuzzy Numbers

Health risk assessment is one of the significant methods to evaluate the risk of environmental pollution on human beings. However, in practical application, due to the complexity of environmental system and limitations of people’s cognitive level, there are always a large amount of uncertainties in health risk assessment, including parameter uncertainty, scenario uncertainty, and model uncertainty [32]. Triangular fuzzy numbers were introduced into health risk assessment to reduce and quantify parameter uncertainties.

The main research objects in this study were inhabitants around Honghu Lake. Honghu Lake is surrounded by countryside, with the Chatan Peninsula in the center of the lake. Some surrounding farmers and fishermen on the Chatan Peninsula take preliminarily preprocessed water from Honghu Lake as domestic water and drinking water, especially fishermen, who are closely related to Honghu Lake, and drink and use water from it. Sampling time of surface water was determined during August 2016 for a screening-level assessment to preliminarily identify the risk priority pollutants and control areas. There are great individual and regional differences in human exposure characteristic to pollutants. Thus, exposure parameter selection should synthetically consider regional characteristics, occupational features, season and gender. On the basis of considering various factors, the exposure parameters are no longer single constants. Therefore, triangular fuzzy numbers are applied on selection of exposure parameters accordingly, combining with α-cut, and transform the exposure parameters involved into intervals for health risk assessment.

There are differences among different individuals in the selection of exposure parameters, and these parameters tend to obey the Gauss distribution or the approximate Gauss distribution. Triangular fuzzy numbers can approximately fit the Gauss distribution. Triangular fuzzy numbers have good applicability to deal with data which is lack of insufficient information or accuracy. It synthetically considers several factors in parameter selection, and describes the randomness and fuzziness of the parameters by the membership function, defined as follows: Assuming that there is a fuzzy number *Ã* in the real number R, define a membership function [33]:

(7) $$\mu_{\tilde{A}}\left(x\right)=\{\begin{array}{cc} 0 & x<a_{1}\\ \frac{x-a_{1}}{a_{2}-a_{1}} & a_{1}\leq x\leq a_{2}\\ \frac{a_{3}-x}{a_{3}-a_{2}} & a_{2}\leq x\leq a_{3}\\ 0 & x>a_{3} \end{array}$$

Then *Ã* is described as triangular fuzzy numbers, and recorded as *Ã* = (*a*_1_, *a*_2_, *a*_3_), where *a*_1_, *a*_2_, *a*_3_ are the minimum value, the most possible value, and the maximum value, respectively (*a*_1_ ≤ *a*_2_ ≤ *a*_3_). According to mathematical statistics methods and numerical analysis principle [34], the choosing method of *a*_1_, *a*_2_, *a*_3_ is as follows: *a*_1_ picks the larger value after comparing the minimum value and (Mean − 2SD) of the data; *a*_2_ is the statistical expectation of the data, which reflects overall size feature of the random variables. The commonly-used statistics of statistical expectation include the arithmetic mean, geometric mean and median, and the final selection depends on the distribution characteristics of the random variables; *a*_3_ picks the smaller value after comparing the maximum value and (Mean + 2SD) of the data.

The membership degree of triangular fuzzy numbers represents the reliability of each data point in the interval of the minimum possible value and maximum possible value. Different confidence reliabilities α (0 < α < 1) correspond to different intervals. This means when *Ã*_α_ = {x|μ*_Ã_*(x) > α, x ∈ X}, and *Ã*_α_ is an α–cut of *Ã*, which represents the data set with reliability not less than α, then *Ã*_α_ = [*a*_s_^α^, *a*_b_^α^] = [α(*a*_2_ − *a*_1_) + a_1_, −α(*a*_3_ − *a*_2_) + *a*_3_] [35]. In general, when α ≥ 0.9, the interval obtained is of high reliability, which is more conducive for managers to control the risk [36].

Finally, the health risk level posed by trace elements is identified by membership function calculation based on Table 1. Assuming that there is an interval of risk [CR_1_, CR_2_], then the membership degree of [CR_1_, CR_2_] in [CR_1_*, CR_2_*] can be quantified as [33]:

(8) $$A\left(\lambda \right)=\frac{\left|\left[{\mathrm{CR}}_{1}{,\mathrm{CR}}_{2}\right]\cap {[\mathrm{CR}}_{1}^{*}{,\mathrm{CR}}_{2}^{*}]\right|}{\left|{[\mathrm{CR}}_{1}{,\mathrm{CR}}_{2}]\right|}$$

where A(λ) represents the membership degree of [CR_1_, CR_2_] in [CR_1_*, CR_2_*]; “| |” represents the geometric length of intervals; “∩” represents taking the intersection of two intervals; and [CR_1_*, CR_2_*] represents the Grade λ of the risk ranks, λ = 1, 2, 3, …, 7.

In this study, the selection of exposure parameters mainly referred to “Exposure Factors Handbook of Chinese Population (Adult volume)” issued by Chinese Ministry of Environmental Protection. Based on a comprehensive consideration of region, occupation, season, gender, and actual situation of local fishermen, the values of IR, BW, and SA were determined, and the triangular fuzzy numbers were taken as IR = (0.46, 1.98, 3.5) L/day, BW = (52.28, 1.5, 72.63) kg, SA = (1.5, 1.68, 1.85) m^2^, respectively [37,38].

Exposure duration (ED) is defined as “length of time over which contact with the contaminant lasts” by USEPA [39]. In “Guidelines for Exposure Assessment” posed by USEPA, the recommended value of exposure duration for adults is 30 years, which is mainly considering occupational exposure. Since then, the value of ED in many studies referred to the recommended value of USEPA. However, some scholars proposed that if the human exposure to pollutants started from birth and occurred every day, the life expectancy should be selected as the exposure duration [40]. Honghu Lake, the study area of this research, is not only an aquaculture area, but also a national nature reserve. Therefore, the evaluation objects include inhabitants surrounding the lake, residents on Chatan peninsula, and managers of the reserve. Obviously, the exposure duration of different objects has wide differences. Considering the recommended value of USEPA and the actual situation in Honghu Lake, the interval of ED was selected as (30, AT) years.

The carcinogenic and non-carcinogenic risks of trace elements were different in the values of average time (AT) when calculating the exposure dose. For non-carcinogenic risk, the average time is equal to the exposure duration, i.e., AT = ED × 365; but for carcinogenic risk, the risk exists in the entire life of the exposure objects, so average time is the average life expectancy of people. In this study, AT referred to the average life expectancy of residents in Hubei Province [37], and the triangular fuzzy numbers of average life was AT = (24,271, 26,678, 29,085) days.

The triangular fuzzy numbers of exposure parameters were transformed into intervals with Equation (7) (Table 2).

### 2.4. Multivariate and Geostatistical Methods

SPSS software was applied for data entry and data computation. Basic statistical parameters such as range, mean, medium and standard deviation (SD) were calculated in order to analyze characterization of trace elements. Geographic information system (GIS) was used to present the spatial distribution of trace elements and health risk level in Honghu Lake [41]. The inverse distance weighted (IDW) method was applied to map the spatial distribution of pollutants based on the ArcGIS 9.3 software (ArcGIS 9.3, Environmental Systems Research Institute Inc., Redlands, CA, USA). IDW can carry out the spatial analysis for points which is relatively independent of the surrounding data points, and does not induce the smoothing effect.

## 3. Results and Discussion

### 3.1. Basic Parameters and Trace Element Concentrations in Surface Water from Honghu Lake

**Figure 2:**
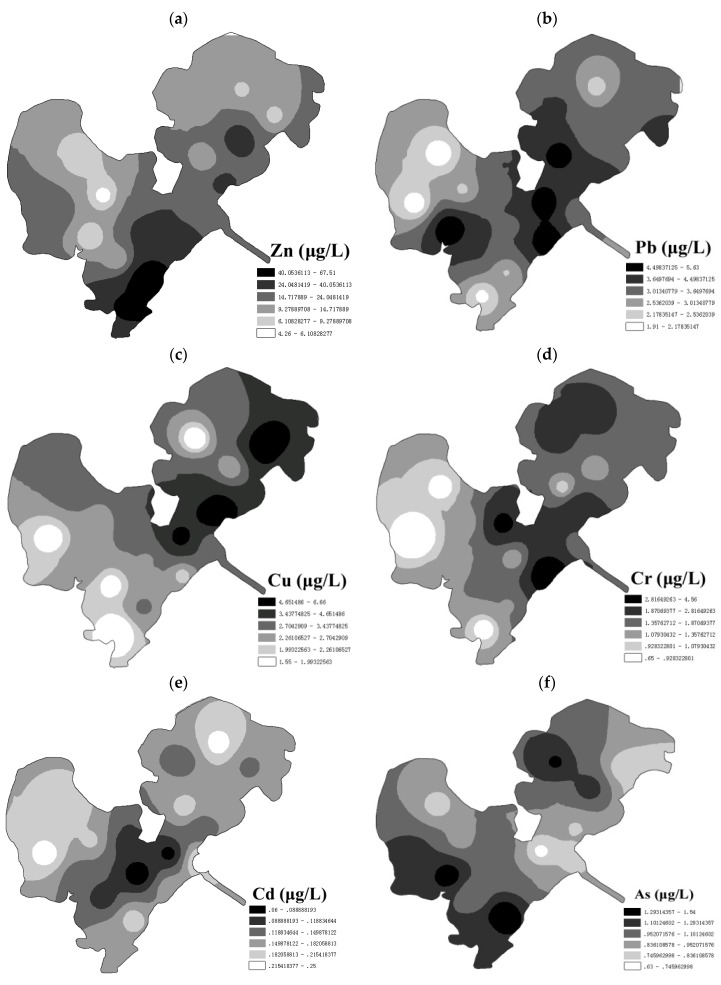
Spatial distribution of Zn (**a**), Pb (**b**), Cu (**c**), Cr (**d**), Cd (**e**) and As (**f**) in surface water from Honghu Lake.

The pH, dissolved oxygen (DO), electrical conductivity (EC), and total amount of trace element in surface water from Honghu Lake are shown as Table 3 and Figure 2. The range of pH was from 7.26 to 7.79, with the mean value of 7.59. The variation of water pH in Honghu Lake was not notable, and pH of each sampling site was within the permissible limits of Drinking Water Guidelines of China, World Health Organization (WHO), and USEPA [42,43,44]. The DO in surface water was in the range of 6.34 to 12.42 mg O_2_/L, which had great difference among different sampling sites. As shown in Table 3, the maximum DO content was twice the lowest value. The average DO content was 9.47 mg O_2_/L, which reached the Class I (7.5 mg O_2_/L) of surface water standard [45]. The conductivity of water was between 230 μs/cm to 356 μs/cm, with the mean value of 275.25 μs/cm, and was within the permissible limits of WHO drink water guidelines. There is no direct regulation on the conductivity of surface water and drinking water in China, so the conductivity has to been roughly estimated through the limits of total dissolved solids (TDS) and total hardness [42]. From the physicochemical indexes, water quality of Honghu Lake met the national drinking water standard.

Mean total contents of Zn, Cu, Cd, Cr, As and Pb in the surface water were 20.45, 3.09, 0.14, 1.63, 0.99 and 3.42 μg/L, respectively, which decreased in the order of Zn > Pb > Cu > Cr > As > Cd. The concentrations of all selected trace elements were within the permissible limits of China [41], WHO and USEPA. Average content of Zn was the highest, with the range of 4.26–67.51 μg/L, which indicated significant fluctuation of Zn concentration in surface water samples from various sites. According to Figure 2a, concentration of Zn in surface water decreased in the order of S4 > S13 > S15 > S18 > S2 > S16 > S20 > S3 > S11 > S10 > S6 > S19 > S17 > S7 > S8 > S1 > S12 > S9 > S14 > S5, so the sites of higher Zn concentrations were in the south of Honghu Lake.

The concentration of Pb was second, followed by Cu, with average concentrations of 3.42 μg/L and 3.09 μg/L, respectively. Concentrations of Pb increased in the order of S9 ≤ S11 < S13 < S8 < S14 < S4 < S10 < S18 < S17 < S6 < S1 < S15 < S19 < S12 < S20 < S2 < S7 < S3 < S16 < S5 (Figure 2b). By contrast, the areas of relatively higher concentrations of Cu were around S1, S2 and S3 (Figure 2c). Figure 2d shows the spatial distribution of Cr, which illustrated the descending order of S16 > S6 > S2 > S19 > S8 > S3 > S1 > S17 > S12 > S20 > S4 > S10 > S5 > S18 > S14 > S15 > S7 > S9 > S13 > S11. Figure 2b–d,f show that sites of higher concentrations of Pb, Cu, Cr and Cd were around Chatan Peninsula, which indicating disturbance of anthropogenic activities in Chatan Peninsula.

Average contents of As from each sampling site decreased in the order of S4 > S5 > S19 > S18 > S13 > S11 > S6 > S8 > S15 > S7 > S16 > S10 > S14 > S12 > S2 > S9 > S17 > S3 > S1 > S20. Figure 2f shows that northeast and west part of Honghu Lake were of higher As concentration. Northeast of the lake is one of inflow entrance areas, and west of the lake is the aquaculture area, which indicated that As in Honghu Lake may originate mainly from upstream pollution and aquaculture.

To better understand the situation of trace elements in surface water from Honghu Lake, contents of selected trace elements were compared with published data of other freshwater lakes at home and abroad (Table 4). The results illustrated that trace elements in surface water from Honghu Lake in 2016 were of a relatively severer pollution degree than that in 2012, except Cr and As. Honghu Lake is connected with the Yangtze River by a number of culverts and sluices, which plays a key role in water storage. However, as the “water bag” of the middle Yangtze River, trace element concentrations in Honghu Lake were much lower than that in the Yangtze River except Zn. Obviously, the concentration of Zn in Honghu Lake was higher than most of the other freshwater lakes, but it still did not exceed Xiangjiang River and Tigris River. In addition, concentration of As in Honghu Lake was relatively lower than other lakes, especially the Yangtze River, the upper Han River, and Xiangjiang River.

### 3.2. Fuzzy Health Risks Assessment for Exposure to Trace Elements

Carcinogenic risk and non-carcinogenic risk values of selected trace elements via ingestion and dermal absorption were calculated by Equations (1)–(6), and the results are presented in Table 5 and Table 6.

#### 3.2.1. Non-Carcinogenic Risk of Trace Elements on Receptors

Table 5 shows non-carcinogenic risk values of selected trace elements based on fuzzy health risk assessment model and certainty assessment model.

Non-carcinogenic risk levels based on fuzzy assessment were basically consistent with the results based on certain assessment. Average levels of non-carcinogenic risk in surface water were observed in the descending order of 1 > As > Pb > Cr > Cd > Zn > Cu. Hazard index (HI) of selected trace elements was below unity, which means no obvious non-carcinogenic hazard to human. However, the range of non-carcinogenic risks based on fuzzy evaluation was greater than that based on certain evaluation owing to a wider selection of parameters. Health risk assessment based on triangular fuzzy numbers can contain more information on contaminant concentrations and exposure parameters, and reflect the uncertainty caused by spatial and individual difference to some extent.

Cr, As and Pb were the major contributors of non-carcinogenic risk on receptors, which accounted for more than 90% of the total non-carcinogenic risk. Hazard quotient (HQ) of As was the highest, which accounted for 60% of the integrated non-carcinogenic risk on average. The main source of As in Honghu Lake was probably artificial activities such as pesticides and fertilizers. Furthermore, HQ via ingestion was 1–2 orders of magnitude higher than HQ via dermal contact (Tables S1 and S2). It indicated that ingestion of drinking water was the primary exposure pathway for residents around Honghu Lake, which was consistent with the results in previous studies [20,46].

Spatially, the hazard index (HI) via ingestion and dermal absorption of selected trace elements from sampling sites decreased in the order of S5 > S16 > S4 > S19 > S6 > S2 > S18 > S13 > S15 > S7 > S3 > S8 > S11 > S12 > S1 > S10 > S14 > S17 > S20 > S9 (Table S3). Higher non-carcinogenic risk of trace elements in S4, S5, and S9 was caused by As, while higher non-carcinogenic risk in S6 and S16 seemed to be caused by Cr. Although the results indicated that there was no obvious non-carcinogenic risk observed among selected trace elements, it still needed to be under routine monitoring and attention paid to it. On one hand, limited by the experimental conditions, there were only six representative trace elements selected in this study, and other trace elements were not taken into consideration. On the other hand, sampling time of the study was selected in late August for a screening level assessment, and previous research had shown that concentrations of trace elements in surface water in winter was higher than that in summer. This means that the risk value in winter maybe higher than that in summer [22]. Subsequent research will be appropriately focused based on the evaluation results of this study.

#### 3.2.2. Carcinogenic Risk of Trace Elements on Receptors

There are four kinds of trace elements with carcinogenic risk among selected trace elements: Cd, Cr, As and Pb. The exposure pathways of human exposing to Cd and As in water were identified including ingestion and dermal contact, while that of Cr and Pb were identified only via ingestion.

Table 6 indicated that integrated carcinogenic risk was observed in the descending order of As > Cr > Cd > Pb. Similarly, As was regarded as the major contributor of carcinogenic risk because it accounted for about 64% of the total carcinogenic risks on average. It has been widely accepted that low exposure dose of As can cause cancer [50]. Therefore, even if the concentration of As in surface water does not exceed the standards, long-term human exposure to As may lead to carcinogenic risk. Carcinogenic risk of Cr ranked in second place among the four trace elements, which was caused by strong toxicity.

The same as non-carcinogenic risk, carcinogenic risk of the same trace elements concentration through drinking water was 1–2 orders of magnitude higher than that through dermal absorption (Table 6). Spatially, integrated carcinogenic risk of selected trace elements from sampling sites decreased in the order of S16 > S6 > S19 > S4 > S5 > S2 > S8 > S18 > S13 > S3 > S11 > S12 > S15 > S10 > S1 > S7 > S17 > S14 > S20 > S9, which was relatively consistent with the results of non-carcinogenic risk (Table S4). The highest carcinogenic risk value in S16 was about twice the lowest value in S9. Therefore, though there were differences among carcinogenic risk values at different sampling sites, they were basically in the same order of magnitude.

Although carcinogenic risk levels based on fuzzy health risk assessment were basically consistent with the results based on certain assessment, there was also sort of difference in some places. For instance, average carcinogenic risk of selected trace elements based on certain assessment was within the target risk posed by USEPA and ICRP. However, when carcinogenic risk was calculated based on the fuzzy health risk assessment model, carcinogenic risk exposed by As exceeded the target risk of ICRP. Furthermore, maximum carcinogenic risk values in S4, S6, S16, and S19 went beyond the target risk of USEPA (Table S4), while carcinogenic risk values based on certain assessment were all within the limited value (Table S5). The reason for the result was that the selection range of pollutant concentrations and exposure parameters for fuzzy assessment were greater than certain assessments. This was especially the case for exposure duration, as fuzzy assessment considered the possibility of lifelong exposure. Parameters based on triangular fuzzy numbers contained more information than the single exposure parameter, reflecting uncertainty to some extent.

As shown in Table S4, even contents of selected trace elements were within the acceptable limits of drinking water guidelines, but maximum integrated carcinogenic risk on receptors in some sites exceeded the target risk. The reason for the conflict is that selection of exposure parameters in this study took many factors (occupation, region, season, and gender) into account, which makes the exposure dose higher than that of the general population. There is no doubt that drinking water standards are formulated based on a large quantity of spot investigation and scientific research, and the limits of each index are strictly determined. At the moment, however, each index has only one universal limit without distinctiveness. We recommend that standards can be revised to adapt to diverse groups and different periods. There are regionally large differences in water intake of people due to water resources, living environment and living habits. For example, the average daily drinking water intake in Northwest China is 2595 mL/day, while that in Northeast China is only 1551 mL/day [37]. Seasonal difference of water intake is also obvious. Daily water intake in summer is approximately 1.5 times that of winter in the same area. In addition, the dermal contact time and contact area of people with water vary greatly in different seasons. Occupation can cause a large difference in the dependence of people on a certain waters. For example, the fishermen involved in this study were more dependent on Honghu Lake than general population. Therefore, we suggest that drinking water guidelines should consider the diversity of groups.

#### 3.2.3. Probabilistic Integrated Carcinogenic Risk Levels of Trace Elements in Surface Water from Honghu Lake

Based on Equation (8) and Table 1, the reliability degree of carcinogenic risk corresponding to each risk level was obtained. Table 7 shows the subordinate grade and corresponding reliability degree of each trace element. It indicated that health risk posed by Cd in surface water of Honghu Lake had a 29% chance corresponding to Grade I and 71% chance corresponding to Grade II. The evaluation results suggested that there was uncertainty on the judgment of the priority pollutant, which may mislead the final decision.

Spatially, carcinogenic risk of trace elements in S4, S6, 16, and S19, were among Grade III (low-medium risk), Grade IV (medium risk) and Grade V (medium-high risk), while that in other 16 sites was between Grade III (low-medium risk) and Grade IV (medium risk). It proved that there was great fuzziness and uncertainty in the judgment of carcinogenic risk level. Based on maximum membership principle, integrated carcinogenic risk of S1, S3, S7, S9, S10, S11, S12, S14, S15, S17, and S20 belonged to Grade III (low-medium risk), while S2, S4, S5, S6, S8, S13, S16, S18, and S19 were judged as Grade IV (medium risk). The results reflected that trace elements in surface water of most areas in Honghu Lake were approximately at the medium risk level. However, in S4, S6, S16, and S19, the membership degrees of carcinogenic risk in Grade V (medium-high risk) were 0.045, 0.169, 0.339, and 0.094, respectively. It implied risks of the four sites should be paid attention and need to be under relatively frequent monitoring to prevent potential high health risk. In particular, the site S6, which was closer to Chatan Peninsula of Honghu Lake, may endanger the health of residents who depend on surface water from Honghu Lake for potable and domestic use.

**Figure 3:**
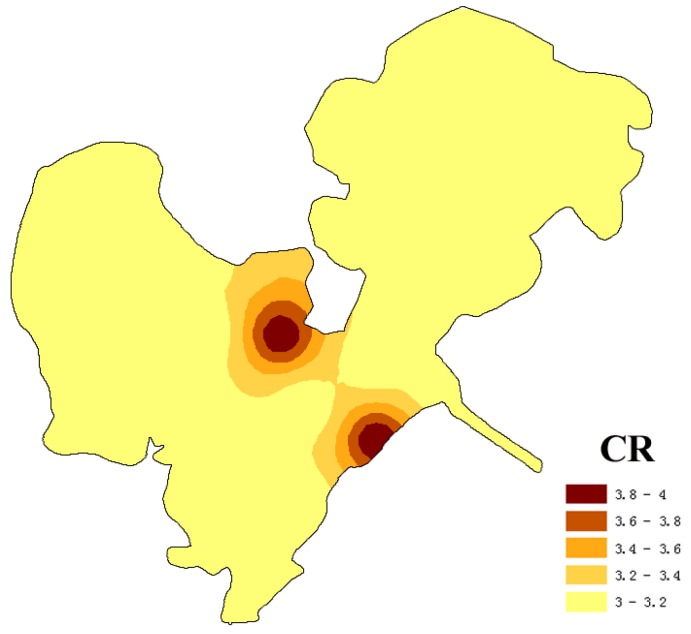
Integrated carcinogenic risk levels based on certainty assessment of surface water from Honghu Lake.

**Figure 4:**
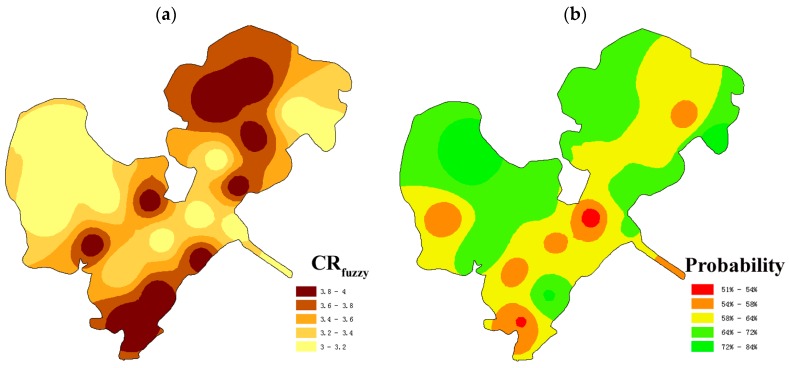
Integrated carcinogenic risk levels based on fuzzy assessment (**a**) and its corresponding probability subjection (**b**) of surface water from Honghu Lake.

Based on maximum membership principle, spatial risk levels of integrated carcinogenic risk under certainty assessment and fuzzy assessment were shown in Figure 3 and Figure 4, respectively. Compared Figure 3 and Figure 4a, the areas under Grade III (low-medium risk) and Grade IV (medium risk) were both 100% of the Honghu Lake, and their spatial risk levels were similar to some extent. From certainty assessment to fuzzy assessment, the original Grade III (low-medium risk) sites (S2, S4, S5, S8, S13, S18, and S19) turned into Grade IV (medium risk) sites, while the other sites kept original risk levels. The cause of rating level based on fuzzy assessment was slightly higher than certainty assessment was that fuzzy assessment considered more factors in the parameter selection. Greater span of parameters in fuzzy assessment contained more information than the statistical average value, which reflected the uncertainty of parameters to a certain extent. Meanwhile, the corresponding reliability degrees at S1, S3, S10, S11, S12, S13, S15 and S18 were of low probability (50–60%) (Figure 4b). The situation that the reliabilities of health risk corresponding to different levels are quite close is likely to provide partial information to decision-makers in the primitively deterministic assessment. Fortunately, health risk assessment based on triangular fuzzy numbers can present the risk level and corresponding reliability, and provide comprehensive information for decision-makers.

The areas in the south (S4, S13, and S16) and northeast (S8, S18, and S19) of Honghu Lake were recommended as the risk priority control areas. Figure S1 presents the surrounding conditions of S8, S13, S16 and S18. This indicated that areas around these sampling sites were enclosed with mesh for aquaculture. Aquaculture activities may raise the contents of trace elements in water and lead to eutrophication. Consequently, the fuzzy health risk assessment, which reflected individual differences more objectively, proved to be of a more suitable method than the certainty assessment.

The fuzzy health risk assessment of trace elements in surface water from Honghu Lake indicated that triangular fuzzy numbers were useful in reducing and expressing the parameter uncertainty. Another two lakes/rivers (Dongting Lake and Pear River Estuary) were selected to further support the conclusion [51]. Evaluation results of trace elements in surface water from Dongting Lake and Pear River Estuary are showed in Tables S6 and S7.

The reliability degrees distribution of Cd and As in Dongting Lake, Cd and Pb in Pear River Estuary, indicated that there was uncertainty on the judgment of risk level, which may mislead final decisions. Especially, reliability degrees of carcinogenic risk posed by Pb in Pear River Estuary were quite close in Grade I (0.42) and Grade II (0.58), and it may mislead the determination on judgment of the priority pollutant. As Table S7 shown, integrated carcinogenic risk level of trace elements in Dongting Lake and Pear River Estuary based on certainty and fuzzy health risk assessment were relatively similar. However, the corresponding reliabilities of carcinogenic risk level in Yueyang Tower and Outlet of Dongting Lake corresponding to Grade V were of low probability (50–60%), and were quite close to the membership to Grade IV. A fairly close case of reliability at different levels may provide one-sided information for decision making. Application of triangular fuzzy numbers in health risk assessment can reduce and quantify the corresponding parameter uncertainty, and provide more comprehensive information for decision-makers.

### 3.3. Uncertainty Analysis

Uncertainty analysis is an important step in health risk assessment. Some uncertainties can be controlled through methodologies, but some are difficult to quantify. Health risk of trace elements on human is dependent on the speciation of elements. Speciation analysis of selected trace elements was not carried out in this study, as it is a screening level assessment, and it will be improved in further study. In addition, owing to non-determination about living conditions of residents around Honghu Lake, the selected exposure pathways may be uncertain to some extent. In this study, two exposure pathways (ingestion and dermal contact) of human exposure to trace elements in surface water were assumed, and As was regarded as the major contributor of health risk. However, with the improvement of people's living standard, drinking water of some residents living around Honghu Lake may come from tap water rather than preliminarily processed water from Honghu Lake. If the water from Honghu Lake is no longer used as drinking water for some residents, it is worth considering whether As is still the risk priority pollutant. Tables S1, S3, and S4 indicated that no matter if residents take the water from Honghu Lake as potable and domestic use, or merely as domestic use, the health risk exposure by As on receptors was the highest among selected trace elements. Though there are some uncertainties, the present study can supply important information for decision-makers to take protective measures and establish management schemes.

## 4. Conclusions

The spatial distribution and screening-level fuzzy health risk assessment of Cu, Zn, Cr, Cd, Pb and As in surface water from Honghu Lake were investigated. The measured levels of trace elements decreased in the order of Zn > Pb > Cu > Cr > As > Cd, within the permissible limits of drinking water criteria recommended by China, USEPA and WHO. Triangular fuzzy numbers were applied in the health risk assessment to reduce parameter uncertainty, and risk classification was carried out to control the uncertainty of risk level judgment. The average levels of non-carcinogenic risk in surface water decreased on the order of 1 > As > Pb > Cr > Cd > Zn > Cu, which means no obvious non-carcinogenic hazard to human health. However, potential carcinogenic risk of trace elements through ingestion and dermal contact was observed in the descending order of As > Cr > Cd > Pb. Integrated carcinogenic risk of trace elements in several sampling sites exceeded the target risk. Even though total contents of selected trace elements were within the acceptable limits, the maximum integrated carcinogenic risk on receptors in some sites exceeded the target risk, so we recommend that standards can be revised to adapt to diverse groups and different periods. The carcinogenic risk of trace elements in S4, S6, S16 and S19 were among Grade III (low-medium risk), Grade IV (medium risk) and Grade V (medium-high risk), while that of other 16 sites was between Grade III (low-medium risk) and Grade IV (medium risk). The result proved that there was great fuzziness in the judgment of carcinogenic risk level, which may mislead the decision-makers’ final judgment. Based on the maximum membership principle, integrated carcinogenic risk of 11 sites was judged to correspond to Grade III, and the other nine sites were judged to correspond to Grade IV. Rating level based on fuzzy assessment was slightly higher than certainty assessment because fuzzy assessment considered more factors in the parameter selection. As was finally regarded as the major contributor of health risk, and areas in the south (S4, S13, and S16) and northeast (S8, S18, and S19) of Honghu Lake were regarded as the risk priority control areas. In order to guarantee the health of residents around Honghu Lake, it is necessary to regularly monitor the water quality in Honghu Lake and evaluate the health risk posed by trace elements.

## Figures and Tables

**Table 1:** Levels and values of assessment standards.

Risk Grades		Range of Risk Value	Acceptability
Grade I	Extremely low risk	<10^−6^	Completely accept
Grade II	Low risk	(10^−6^, 10^−5^)	Not willing to care about the risk
Grade III	Low-medium risk	(10^−5^, 5 × 10^−5^)	Do not mind about the risk
Grade IV	Medium risk	(5 × 10^−5^, 10^−4^)	Care about the risk
Grade V	Medium-high risk	(10^−4^, 5 × 10^−4^)	Care about the risk and willing to invest
Grade VI	High risk	(5 × 10^−4^, 10^−3^)	Pay attention to the risk and take action to solve it
Grade VII	Extremely high risk	>10^−3^	Reject the risk and must solve it

**Table 2:** Exposure parameters treated by α-cut.

IR (L/day)	BW (kg)	SA (m^2^)	ED (Year)	AT (Day)
(1.83, 2.13)	(60.66, 62.52)	(1.66, 1.70)	(30, 73.75)	(26,437, 26,919)

**Table 3:** Summary statistics of basic indexes and total content of trace elements in surface water samples (*N* = 20) from Honghu Lake.

Parameters	pH	DO (mg O_2_/L)	EC (μS/cm)	Total Content of Trace Elements (μg/L)					
				Zn	Cu	Cd	Cr	As	Pb
Mean	7.59	9.47	275.25	20.45	3.09	0.14	1.63	0.99	3.42
Max	7.79	12.42	356	67.51	6.66	0.25	4.56	1.54	5.63
Min	7.26	6.34	230	4.26	1.55	0.06	0.65	0.63	1.91
SD	0.16	1.89	33.88	17.1	1.46	0.05	0.97	0.25	1.15
Detection limits				5	1	0.05	0.1	0.05	0.44
N (%)	100	100	100	90	100	100	100	100	100
WHO [43]	6.5–8.5		1500	3000	2000	3	50	10	10
USEPA [44]	6.5–8.5			5000	1300	5	100	10	15
Chinese standards [42]	6.5–8.5		2000	1000	1000	5	50	10	10

**Table 4:** Summaries of measured trace elements in freshwater from freshwater lakes at home and abroad (μg/L).

Name of Lakes	Zn	Cu	Cd	Cr	As	Pb	Reference
Honghu Lake, China	2.13	1.93	0.04	1.71	2.83	1.28	[13]
The Yangtze River, China	9.40	10.70	4.70	20.90	13.20	55.10	[46]
The upper Han River, China	NA	21.65	3.78	-	20.05	2.31	[47]
East Dongting Lake, China	8.86	0.07	0.05	-	3.23	0.04	[2]
Xiangjiang River, China	84.57	20.33	1.34	6.61	12.24	2.29	[3]
Rawal Lake, Pakistan	14	10	6	9	-	162	[22]
Catalan River, Spain	1.9	1.3	1.2	2.4	2.9	2.2	[48]
Tigris River, Turkey	37	165	1.37	<5	2.35	0.34	[49]
This study	18.04	3.09	0.14	1.63	0.99	3.42	

**Table 5:** Hazard quotient (HQ) and hazard index (HI) of trace elements in surface water from Honghu Lake.

Assessment Methods	HQ						HI	Target Risk
	Zn	Cu	Cd	Cr	As	Pb		
Fuzzy assessment	(1.93 × 10^−3^, 2.43 × 10^−3^)	(2.18 × 10^−3^, 2.61 × 10^−3^)	(5.85 × 10^−3^, 6.81 × 10^−3^)	(2.35 × 10^−2^, 2.72 × 10^−2^)	(9.37 × 10^−2^, 1.14 × 10^−1^)	(2.73 × 10^−2^, 3.32 × 10^−2^)	(1.52 × 10^−1^, 1.86 × 10^−1^)	1 [26]
Certain assessment	2.13 × 10^−3^	2.42 × 10^−3^	6.28 × 10^−3^	2.53 × 10^−2^	1.03 × 10^−1^	3.02 × 10^−2^	1.70 × 10^−1^	

**Table 6:** Carcinogenic risk (CR) of trace elements in surface water from Honghu Lake.

Assessment Methods	Cd		Cr	As		Pb	CR	Target Risk
	CR_ing_	CR_derm_	CR_ing_	CR_ing_	CR_derm_	CR_ing_		
Fuzzy assessment	(5.86 × 10^−7^, 1.75 × 10^−6^)	(5.12 × 10^−8^, 1.34 × 10^−7^)	(9.28 × 10^−6^, 2.76 × 10^−5^)	(1.69 × 10^−5^, 5.06 × 10^−5^)	(2.25 × 10^−7^, 5.90 × 10^−7^)	(3.20 × 10^−7^, 9.85 × 10^−7^)	(2.74 × 10^−5^, 8.16 × 10^−5^)	1.00 × 10^−4^ [26]
Certain assessment	1.083 × 10^−6^	8.84 × 10^−8^	1.71 × 10^−5^	1.74 × 10^−5^	2.31 × 10^−7^	6.07 × 10^−7^	3.66 × 10^−5^	5.00 × 10^−5^ [27]

**Table 7:** Reliability degrees of each trace element in surface water from Honghu Lake in different health risk levels.

Elements	Grade I	Grade II	Grade III	Grade IV	Grade V	Grade VI	Grade VII
Cd	0.29	0.71					
Cr		0.04	0.96				
As			0.97	0.03			
Pb	1						

## Supplementary Materials

The following are available online at www.mdpi.com/1660-4601/14/9/1011/s1, Figure S1: Pictures of surrounding conditions in sites S8 (a), S13 (b), S16 (c, d) and S18 (e), Table S1: Hazard quotient (HQ) and Hazard index (HI) of trace elements in surface water from Honghu Lake through ingestion, Table S2: Hazard quotient (HQ) and Hazard index (HI) of trace elements in surface water from Honghu Lake through dermal contact, Table S3: Hazard quotient (HQ) and Hazard index (HI) of trace elements in surface water from Honghu Lake based on fuzzy assessment, Table S4: Carcinogenic risk (CR) of trace elements in surface water from Honghu Lake based on fuzzy assessment, Table S5: Carcinogenic risk of trace elements in surface water from Honghu Lake based on certainty assessment, Table S6: Reliability degrees of carcinogenic risk caused by trace element in different risk levels, Table S7: Differences of carcinogenic risk level in each sampling sites between fuzzy and certainty assessment.
