# Supplementary material for: Spatial Distribution and Fuzzy Health Risk Assessment of Trace Elements in Surface Water from Honghu Lake
Source: Int J Environ Res Public Health. 2017 Sep 4;14(9):1011. doi: 10.3390/ijerph14091011 (PMC5615548; doi:10.3390/ijerph14091011)
Supplement: Supplementary file 1 [file ijerph-14-01011-s001.pdf]

**Why sampling time was confirmed during August, 2016**

Firstly, Honghu Lake is an important storage lake in Jianghan Plain. It is a semi−closed lake, and the inflows are mainly from the north through the Four−lake Main Canal. Outflow of Honghu Lake discharges into the Yangtze River through the only water gate in southeast, and the water gate is open only in flood period (generally in some days during June−August). Secondly, the rainfall in Honghu Basin is mainly concentrated in summer. The surface runoff in the area flows into Honghu Lake through the Four−lake Main Canal. Upstream water will also bring a large amount of pollutants into Honghu Lake, so the water pollution in flood period is more complicated than other seasons. Finally, during the flood period, Honghu Lake irregularly releases water as water levels rise. August is the end of the flood season, with relatively small fluctuations in water quality. Furthermore, the high water level in Honghu Lake during the flood season will bring inconvenience and danger to the sampling work. So at the preparation stage, we had a close communication with the local guide, environmental protection department and water conservancy department to keep abreast of the latest situation in Honghu Lake. We confirmed the sampling time at the end of the August when was about two weeks from the last water releasing time. Therefore, in our sampling date, the lake came into a relatively stable state. Based on the above, sampling time in this study was determined at the end of the August.

**Table S1.** Hazard quotient (HQ) and Hazard index (HI) of trace elements in surface water from Honghu Lake through ingestion.

| Sites | $HQ_{ing}$ | | | | | | $HI_{ing}$ |
|---|---|---|---|---|---|---|---|
| | Zn | Cu | Cd | Cr | As | Pb | |
| S1 | [6.82E−04,8.25E−04] | [4.46E−03,5.45E−03] | [4.52E−03,5.43E−03] | [1.59E−02,2.00E−02] | [6.62E−02,8.69E−02] | [2.68E−02,3.34E−02] | [1.19E−01,1.52E−01] |
| S2 | [2.54E−03,3.10E−03] | [4.62E−03,5.66E−03] | [4.95E−03,6.27E−03] | [2.69E−02,3.23E−02] | [7.63E−02,9.22E−02] | [3.45E−02,4.14E−02] | [1.50E−01,1.81E−01] |
| S3 | [2.07E−03,2.52E−03] | [3.54E−03,4.43E−03] | [6.47E−03,7.87E−03] | [1.80E−02,2.16E−02] | [6.65E−02,8.06E−02] | [4.05E−02,4.89E−02] | [1.37E−01,1.66E−01] |
| S4 | [6.30E−03,7.59E−03] | [1.94E−03,2.38E−03] | [2.81E−03,3.46E−03] | [1.23E−02,1.48E−02] | [1.44E−01,1.73E−01] | [1.99E−02,2.41E−02] | [1.87E−01,2.25E−01] |
| S5 | [3.97E−04,4.81E−04] | [1.84E−03,2.29E−03] | [5.16E−03,6.19E−03] | [1.03E−02,1.24E−02] | [1.32E−01,1.62E−01] | [4.45E−02,5.50E−02] | [1.94E−01,2.38E−01] |
| S6 | [1.43E−03,1.75E−03] | [2.36E−03,2.83E−03] | [5.60E−03,6.73E−03] | [3.04E−02,3.68E−02] | [9.81E−02,1.18E−01] | [2.69E−02,3.31E−02] | [1.65E−01,1.99E−01] |
| S7 | [8.97E−04,1.08E−03] | [2.42E−03,3.00E−03] | [2.68E−03,3.25E−03] | [8.69E−03,1.05E−02] | [8.82E−02,1.06E−01] | [3.94E−02,4.75E−02] | [1.42E−01,1.72E−01] |
| S8 | [7.66E−04,9.53E−04] | [2.02E−03,2.44E−03] | [2.07E−03,2.57E−03] | [1.91E−02,2.29E−02] | [9.08E−02,1.10E−01] | [1.85E−02,2.25E−02] | [1.33E−01,1.61E−01] |
| S9 | [6.16E−04,7.61E−04] | [2.33E−03,2.80E−03] | [2.32E−03,2.78E−03] | [7.86E−03,9.44E−03] | [7.29E−02,8.85E−02] | [1.53E−02,1.85E−02] | [1.01E−01,1.23E−01] |
| S10 | [1.63E−03,1.98E−03] | [2.29E−03,2.77E−03] | [3.27E−03,3.98E−03] | [1.20E−02,1.46E−02] | [8.36E−02,1.04E−01] | [2.06E−02,2.50E−02] | [1.23E−01,1.52E−01] |
| S11 | [1.88E−03,2.30E−03] | [1.18E−03,1.49E−03] | [2.09E−03,2.51E−03] | [6.04E−03,7.30E−03] | [1.09E−01,1.32E−02] | [1.52E−02,1.85E−02] | [1.36E−01,1.64E−01] |
| S12 | [6.61E−04,7.98E−04] | [1.21E−03,1.48E−03] | [3.92E−03,4.73E−03] | [1.29E−02,1.57E−02] | [8.19E−02,9.93E−02] | [2.98E−02,3.63E−02] | [1.30E−01,1.58E−01] |
| S13 | [3.87E−03,4.72E−03] | [1.07E−03,1.35E−03] | [3.27E−03,4.04E−03] | [7.39E−03,8.95E−03] | [1.12E−01,1.36E−01] | [1.64E−02,1.99E−02] | [1.44E−01,1.75E−01] |
| S14 | [3.99E−04,4.94E−04] | [1.61E−03,2.02E−03] | [3.09E−03,3.75E−03] | [9.68E−03,1.16E−02] | [8.24E−02,9.97E−02] | [1.94E−02,2.36E−02] | [1.17E−01,1.41E−01] |
| S15 | [3.78E−03,4.56E−03] | [1.84E−03,2.24E−03] | [6.93E−03,8.34E−03] | [9.15E−03,1.10E−02] | [9.01E−02,1.08E−01] | [2.80E−02,3.37E−02] | [1.40E−01,1.68E−01] |
| S16 | [2.48E−03,3.03E−03] | [1.45E−03,1.74E−03] | [3.28E−03,4.09E−03] | [4.26E−02,5.12E−02] | [8.79E−02,1.08E−01] | [4.16E−02,5.07E−02] | [1.79E−01,2.19E−01] |
| S17 | [1.17E−03,1.43E−03] | [1.69E−03,2.07E−03] | [1.61E−03,1.93E−03] | [1.34E−02,1.61E−02] | [7.18E−02,8.66E−02] | [2.36E−02,2.89E−02] | [1.13E−01,1.37E−01] |
| S18 | [2.97E−03,3.63E−03] | [1.45E−03,1.80E−03] | [3.21E−03,3.95E−03] | [1.00E−02,1.21E−02] | [1.13E−01,1.36E−01] | [2.23E−02,2.69E−02] | [1.53E−01,1.85E−01] |
| S19 | [1.17E−03,1.43E−03] | [1.07E−03,1.31E−03] | [4.84E−03,5.82E−03] | [1.94E−02,2.33E−02] | [1.24E−01,1.50E−01] | [2.90E−02,2.66E−02] | [1.79E−01,2.18E−01] |
| S20 | [2.30E−03,2.77E−03] | [2.42E−03,2.94E−03] | [3.69E−03,4.48E−03] | [1.23E−02,1.48E−02] | [5.83E−02,7.08E−02] | [3.11E−02,3.79E−02] | [1.10E−01,1.34E−01] |

**Table S2.** Hazard quotient (HQ) and Hazard index (HI) of trace elements in surface water from Honghu Lake through dermal contact.

| Sites | $HQ_{derm}$ | | | | | | $HI_{derm}$ |
|---|---|---|---|---|---|---|---|
| | Zn | Cu | Cd | Cr | As | Pb | |
| S1 | [1.11E−05,1.18E−05] | [8.11E−05,8.69E−05] | [2.46E−03,2.59E−03] | [8.66E−03,9.58E−03] | [8.80E−04,1.01E−03] | [9.73E−05,1.06E−04] | [1.22E−02,1.34E−02] |
| S2 | [4.16E−05,4.45E−05] | [8.40E−05,9.02E−05] | [2.70E−03,3.00E−03] | [1.46E−02,1.55E−02] | [1.01E−03,1.07E−03] | [1.25E−04,1.32E−04] | [1.86E−02,1.98E−02] |
| S3 | [3.39E−05,3.62E−05] | [6.43E−05,7.06E−05] | [3.52E−03,3.76E−03] | [9.79E−03,1.03E−02] | [8.83E−04,9.40E−04] | [1.47E−04,1.56E−04] | [1.44E−02,1.53E−02] |
| S4 | [1.03E−04,1.09E−04] | [3.53E−05,3.80E−05] | [1.53E−03,1.65E−03] | [6.69E−03,7.06E−03] | [1.91E−03,2.02E−03] | [7.24E−05,7.68E−05] | [1.03E−02,1.10E−02] |
| S5 | [6.49E−06,6.90E−06] | [3.34E−05,3.66E−05] | [2.81E−03,2.96E−03] | [5.61E−03,5.95E−03] | [1.76E−03,1.89E−03] | [1.62E−04,1.75E−04] | [1.04E−02,1.10E−02] |
| S6 | [2.33E−05,2.51E−05] | [4.28E−05,4.50E−05] | [3.05E−03,3.22E−03] | [1.66E−02,1.76E−02] | [1.30E−03,1.38E−03] | [9.79E−05,1.06E−04] | [2.11E−02,2.23E−02] |
| S7 | [1.47E−05,1.55E−05] | [4.40E−05,4.79E−05] | [1.46E−03,1.55E−03] | [4.74E−03,5.03E−03] | [1.17E−03,1.24E−03] | [1.43E−04,1.51E−04] | [7.57E−03,8.04E−03] |
| S8 | [1.25E−05,1.37E−05] | [3.67E−05,3.88E−05] | [1.13E−03,1.23E−03] | [1.04E−02,1.10E−02] | [1.21E−03,1.28E−03] | [6.73E−05,7.17E−05] | [1.28E−02,1.36E−02] |
| S9 | [1.01E−05,1.09E−05] | [4.24E−05,4.46E−05] | [1.26E−03,1.33E−03] | [4.28E−03,4.54E−03] | [9.68E−04,1.03E−03] | [5.56E−05,5.89E−05] | [6.62E−03,6.99E−03] |
| S10 | [2.66E−05,2.84E−05] | [4.16E−05,4.41E−05] | [1.78E−03,1.90E−03] | [6.56E−03,7.00E−02] | [1.11E−03,1.21E−03] | [7.47E−05,7.97E−05] | [9.59E−03,1.03E−02] |
| S11 | [3.07E−05,3.30E−05] | [2.14E−05,2.38E−05] | [1.14E−03,1.20E−03] | [3.29E−03,3.49E−03] | [1.45E−03,1.54E−03] | [5.54E−05,5.89E−05] | [5.99E−03,6.34E−03] |
| S12 | [1.08E−05,1.14E−05] | [2.19E−05,2.36E−05] | [2.14E−03,2.26E−03] | [7.04E−03,7.50E−03] | [1.09E−03,1.16E−03] | [1.08E−04,1.16E−04] | [1.04E−02,1.11E−02] |
| S13 | [6.33E−05,6.77E−05] | [1.94E−05,2.15E−05] | [1.78E−03,1.93E−03] | [4.03E−03,4.28E−03] | [1.49E−03,1.58E−03] | [5.96E−05,6.35E−05] | [7.44E−03,7.95E−03] |
| S14 | [6.52E−06,7.08E−06] | [2.92E−05,3.22E−05] | [1.68E−03,1.79E−03] | [5.28E−03,5.57E−03] | [1.10E−03,1.16E−03] | [7.06E−05,7.52E−05] | [8.16E−03,8.63E−03] |
| S15 | [6.18E−05,6.53E−05] | [3.34E−05,3.57E−05] | [3.78E−03,3.98E−03] | [4.98E−03,5.26E−03] | [1.20E−03,1.26E−03] | [1.02E−04,1.07E−04] | [1.02E−02,1.07E−02] |
| S16 | [4.06E−05,4.35E−05] | [2.64E−05,2.78E−05] | [1.79E−03,1.95E−03] | [2.32E−02,2.45E−02] | [1.17E−03,1.26E−03] | [1.51E−04,1.62E−04] | [2.64E−02,2.79E−02] |
| S17 | [1.91E−05,2.05E−05] | [3.07E−05,3.29E−05] | [8.77E−04,9.23E−04] | [7.29E−03,7.68E−03] | [9.54E−04,1.01E−03] | [8.57E−05,9.22E−05] | [9.26E−03,9.75E−03] |
| S18 | [4.85E−05,5.21E−05] | [2.64E−05,2.87E−05] | [1.75E−03,1.89E−03] | [5.45E−03,5.77E−03] | [1.50E−03,1.59E−03] | [8.10E−05,8.57E−05] | [8.86E−03,9.41E−03] |
| S19 | [1.92E−05,2.05E−05] | [1.95E−05,2.10E−05] | [2.64E−03,2.78E−03] | [1.06E−02,1.11E−02] | [1.65E−03,1.75E−03] | [1.05E−04,1.16E−04] | [1.50E−02,1.58E−02] |
| S20 | [3.76E−05,3.97E−05] | [4.39E−05,4.69E−05] | [2.01E−03,2.14E−03] | [6.71E−03,7.07E−03] | [7.75E−04,8.26E−04] | [1.13E−04,1.21E−04] | [9.69E−03,1.02E−02] |

**Table S3**. Hazard quotient (HQ) and Hazard index (HI) of trace elements in surface water from Honghu Lake based on fuzzy assessment.

| **Sites** | **HQ** | | | | | | **HI** |
|---|---|---|---|---|---|---|---|
| | **Zn** | **Cu** | **Cd** | **Cr** | **As** | **Pb** | |
| S1 | [6.93E−04,8.37E−04] | [4.55E−03,5.54E−03] | [6.98E−03,8.02E−03] | [2.45E−02,2.96E−02] | [6.71E−02,8.79E−02] | [2.69E−02,3.35E−02] | [1.31E−01,1.65E−01] |
| S2 | [2.59E−03,3.15E−03] | [4.71E−03,5.75E−03] | [7.65E−03,9.26E−03] | [4.15E−02,4.78E−02] | [7.73E−02,9.32E−02] | [3.46E−02,4.16E−02] | [1.68E−01,2.01E−01] |
| S3 | [2.11E−03,2.56E−03] | [3.60E−03,4.50E−03] | [9.99E−03,1.16E−02] | [2.78E−02,3.19E−02] | [6.74E−02,8.15E−02] | [4.07E−02,4.91E−02] | [1.52E−01,1.81E−01] |
| S4 | [6.41E−03,7.70E−03] | [1.98E−03,2.42E−03] | [4.35E−03,5.11E−03] | [1.90E−02,2.18E−02] | [1.46E−01,1.75E−01] | [2.00E−02,2.42E−02] | [1.98E−01,2.36E−01] |
| S5 | [4.03E−04,4.88E−04] | [1.87E−03,2.33E−03] | [7.97E−03,9.15E−03] | [1.59E−02,1.84E−02] | [1.34E−01,1.64E−01] | [4.47E−02,5.52E−02] | [2.05E−01,2.49E−01] |
| S6 | [1.45E−03,1.77E−03] | [2.40E−03,2.87E−03] | [8.65E−03,9.95E−03] | [4.70E−02,5.43E−02] | [9.94E−02,1.19E−01] | [2.70E−02,3.33E−02] | [1.86E−01,2.21E−01] |
| S7 | [9.11E−04,1.10E−03] | [2.47E−03,3.05E−03] | [4.14E−03,4.80E−03] | [1.34E−02,1.56E−02] | [8.94E−02,1.08E−01] | [3.96E−02,4.77E−02] | [1.50E−01,1.80E−01] |
| S8 | [7.79E−04,9.67E−04] | [2.06E−03,2.47E−03] | [3.19E−03,3.79E−03] | [2.95E−02,3.39E−02] | [9.20E−02,1.11E−01] | [1.86E−02.2.26E−02] | [1.45E−01,1.75E−01] |
| S9 | [6.26E−04,7.71E−04] | [2.37E−03,2.84E−03] | [3.58E−03,4.11E−03] | [1.21E−02,1.39E−02] | [7.38E−02,8.95E−02] | [1.54E−02,1.85E−02] | [1.08E−01,1.30E−01] |
| S10 | [1.65E−03,2.01E−03] | [2.33E−03,2.81E−03] | [5.06E−03,5.88E−03] | [1.86E−02,2.16E−02] | [8.47E−02,1.05E−01] | [2.06E−02,2.51E−02] | [1.31E−01,1.62E−01] |
| S11 | [1.91E−03,2.34E−03] | [1.20E−03,1.52E−03] | [3.23E−03,3.71E−03] | [9.33E−03,1.08E−02] | [1.11E−01,1.33E−01] | [1.53E−02,1.86E−02] | [1.42E−01,1.70E−01] |
| S12 | [6.72E−04,8.10E−04] | [1.23E−03,1.51E−03] | [6.05E−03,6.99E−03] | [2.00E−02,2.32E−02] | [8.30E−02,1.00E−01] | [3.00E−02,3.65E−02] | [1.41E−01,1.69E−01] |
| S13 | [3.93E−03,4.79E−03] | [1.09E−03,1.37E−03] | [5.05E−03,5.98E−03] | [1.14E−02,1.32E−02] | [1.14E−01,1.38E−01] | [1.65E−02,2.00E−02] | [1.52E−01,1.83E−01] |
| S14 | [4.05E−04,5.01E−04] | [1.64E−03,2.05E−03] | [4.78E−03,5.54E−03] | [1.50E−02,1.72E−02] | [8.35E−02,1.01E−01] | [1.95E−02,2.37E−02] | [1.25E−01,1.50E−01] |
| S15 | [3.84E−03,4.62E−03] | [1.87E−03,2.27E−03] | [1.07E−02,1.23E−02] | [1.41E−02,1.62E−02] | [9.13E−02,1.09E−01] | [2.81E−02,3.38E−02] | [1.50E−01,1.79E−01] |
| S16 | [2.52E−03,3.07E−03] | [1.48E−03,1.77E−03] | [5.07E−03,6.04E−03] | [6.58E−02,7.57E−02] | [8.91E−02,1.09E−01] | [4.17E−02,5.09E−02] | [2.06E−01,2.46E−01] |
| S17 | [1.19E−03,1.45E−03] | [1.72E−03,2.10E−03] | [2.49E−03,2.86E−03] | [2.07E−02,2.37E−02] | [7.28E−02,8.76E−02] | [2.37E−02,2.90E−02] | [1.23E−01,1.47E−01] |
| S18 | [3.02E−03,3.69E−03] | [1.48E−03,1.83E−03] | [4.96E−03,5.83E−03] | [1.55E−02,1.78E−02] | [1.15E−01,1.38E−01] | [2.24E−02,2.70E−02] | [1.62E−01,1.94E−01] |
| S19 | [1.19E−03,1.45E−03] | [1.09E−03,1.34E−03] | [7.47E−03,8.60E−03] | [2.99E−02,3.44E−02] | [1.26E−01,1.52E−01] | [2.91E−02,3.67E−02] | [1.94E−01,2.34E−01] |
| S20 | [2.34E−03,2.81E−03] | [2.46E−03,2.99E−03] | [5.70E−03,6.62E−03] | [1.90E−02,2.19E−02] | [5.91E−02,7.16E−02] | [3.13E−02,3.80E−02] | [1.20E−01,1.44E−01] |

**Table S4.** Carcinogenic risk (CR) of trace elements in surface water from Honghu Lake based on fuzzy assessment.

| Sites | Cd | | Cr | As | | Pb | CR |
|---|---|---|---|---|---|---|---|
| | $CR_{ing}$ | $CR_{derm}$ | $CR_{ing}$ | $CR_{ing}$ | $CR_{derm}$ | $CR_{ing}$ | |
| S1 | [6.98E−07,2.06E−06] | [6.11E−08,1.58E−07] | [9.69E−06,3.01E−05] | [1.21E−05,3.91E−05] | [1.61E−07,4.56E−07] | [3.24E−07,9.94E−07] | [2.31E−05,7.29E−05] |
| S2 | [7.65E−07,2.38E−06] | [6.69E−08,1.83E−07] | [1.64E−05,4.85E−05] | [1.40E−05,4.15E−05] | [1.86E−07,4.84E−07] | [4.17E−07,1.23E−06] | [3.18E−05,9.42E−05] |
| S3 | [1.00E−06,2.99E−06] | [8.75E−08,2.30E−07] | [1.10E−05,3.24E−05] | [1.22E−05,3.63E−05] | [1.62E−07,1.45E−06] | [4.91E−07,1.45E−06] | [2.49E−05,7.37E−05] |
| S4 | [4.35E−07,1.31E−06] | [3.81E−08,1.01E−07] | [7.49E−06,2.22E−05] | [2.63E−05,7.79E−05] | [3.50E−07,9.08E−07] | [2.41E−07,7.17E−07] | [3.49E−05,1.03E−04] |
| S5 | [7.97E−07,2.35E−06] | [6.97E−08,1.81E−07] | [6.28E−06,1.87E−05] | [2.42E−05,7.29E−05] | [3.22E−07,8.50E−07] | [5.38E−07,1.64E−06] | [3.22E−05,9.66E−05] |
| S6 | [8.66E−07,2.56E−06] | [7.57E−08,1.96E−07] | [1.85E−05,5.51E−05] | [1.80E−05,5.31E−05] | [2.39E−07,6.19E−07] | [3.26E−07,9.86E−07] | [3.80E−05,1.13E−04] |
| S7 | [4.14E−07,1.23E−06] | [3.62E−08,9.47E−08] | [5.30E−06,1.58E−05] | [1.80E−05,4.79E−05] | [2.15E−07,5.59E−07] | [4.77E−07,1.41E−06] | [2.26E−05,6.70E−05] |
| S8 | [3.19E−07,9.75E−07] | [2.79E−08,7.48E−08] | [1.16E−05,3.44E−05] | [1.66E−05,4.94E−05] | [2.21E−07,5.76E−07] | [2.24E−07,6.69E−07] | [2.91E−05,8.61E−05] |
| S9 | [3.58E−07,1.06E−06] | [3.13E−08,8.11E−08] | [4.80E−06,1.42E−05] | [1.33E−05,3.98E−05] | [1.77E−07,4.64E−07] | [1.85E−07,5.49E−07] | [1.89E−05,5.61E−05] |
| S10 | [5.06E−07,1.51E−06] | [4.43E−08,1.16E−07] | [7.34E−06,2.20E−05] | [1.53E−05,4.67E−05] | [2.03E−07,5.44E−07] | [2.49E−07,7.44E−07] | [2.36E−05,7.15E−05] |
| S11 | [3.23E−07,9.55E−07] | [2.83E−08,7.33E−08] | [3.69E−06,1.09E−05] | [2.00E−05,5.93E−05] | [2.66E−07,6.91E−07] | [1.84E−07,5.50E−07] | [2.45E−05,7.25E−05] |
| S12 | [6.06E−07,1.80E−06] | [5.30E−08,1.38E−07] | [7.89E−06,2.35E−05] | [1.50E−05,4.47E−05] | [1.99E−07,5.21E−07] | [3.61E−07,1.08E−06] | [2.41E−05,7.18E−05] |
| S13 | [5.05E−07,1.54E−06] | [4.42E−08,1.18E−07] | [4.51E−06,1.34E−05] | [2.05E−05,6.12E−05] | [2.73E−07,7.13E−07] | [1.99E−07,5.93E−07] | [2.61E−05,7.75E−05] |
| S14 | [4.78E−07,1.42E−06] | [4.18E−08,1.09E−07] | [5.91E−06,1.75E−05] | [1.51E−05,4.49E−05] | [2.01E−07,5.23E−07] | [2.35E−07,7.02E−07] | [2.20E−05,6.51E−05] |
| S15 | [1.07E−06,3.17E−06] | [9.37E−08,2.43E−07] | [5.58E−06,1.65E−05] | [1.65E−05,4.87E−05] | [2.19E−07,5.68E−07] | [3.39E−07,1.00E−06] | [2.38E−05,7.02E−05] |
| S16 | [5.08E−07,1.55E−06] | [4.44E−08,1.19E−07] | [2.60E−05,7.68E−05] | [1.61E−05,4.85E−05] | [2.14E−07,5.66E−07] | [5.03E−07,1.51E−06] | [4.34E−05,1.29E−04] |
| S17 | [2.49E−07,7.34E−07] | [2.18E−08,5.63E−08] | [8.16E−06,2.41E−05] | [1.31E−05,3.90E−05] | [1.75E−07,4.54E−07] | [2.85E−07,8.61E−07] | [2.20E−05,6.51E−05] |
| S18 | [4.97E−07,1.50E−06] | [4.34E−08,1.15E−07] | [6.10E−06,1.81E−05] | [2.07E−05,6.13E−05] | [1.75E−07,7.15E−07] | [2.70E−07,8.00E−07] | [2.79E−05,8.26E−05] |
| S19 | [7.48E−07,2.21E−06] | [6.54E−08,1.70E−07] | [1.18E−05,3.49E−05] | [2.27E−05,6.75E−05] | [3.02E−07,7.88E−07] | [3.51E−07,1.09E−06] | [3.60E−05,1.07E−04] |
| S20 | [5.70E−07,1.70E−06] | [4.99E−08,1.31E−07] | [7.51E−06,2.22E−05] | [1.07E−05,3.19E−05] | [1.42E−07,3.72E−07] | [3.77E−07,1.13E−06] | [1.93E−05,5.74E−05] |

**Table S5.** Carcinogenic risk of trace elements in surface water from Honghu Lake based on certainty assessment.

| Sites | Cd | | Cr | As | | Pb | CR | Risk Level |
|---|---|---|---|---|---|---|---|---|
| | $CR_{ing}$ | $CR_{derm}$ | $CR_{ing}$ | $CR_{ing}$ | $CR_{derm}$ | $CR_{ing}$ | | |
| S1 | 1.29E−06 | 1.05E−07 | 1.79E−05 | 2.24E−05 | 2.78E−07 | 5.98E−07 | 4.26E−05 | III |
| S2 | 1.41E−06 | 1.15E−07 | 3.03E−05 | 1.40E−05 | 1.86E−07 | 7.70E−07 | 4.67E−05 | III |
| S3 | 1.85E−06 | 1.51E−07 | 2.03E−05 | 1.22E−05 | 1.62E−07 | 9.06E−07 | 3.55E−05 | III |
| S4 | 8.03E−07 | 6.57E−08 | 1.38E−05 | 2.63E−05 | 3.50E−07 | 4.45E−07 | 4.18E−05 | III |
| S5 | 1.47E−06 | 1.20E−07 | 1.16E−05 | 2.42E−05 | 3.22E−07 | 9.94E−07 | 3.87E−05 | III |
| S6 | 1.60E−06 | 1.31E−07 | 3.43E−05 | 1.80E−05 | 2.39E−07 | 6.02E−07 | 5.48E−05 | IV |
| S7 | 7.65E−07 | 6.25E−08 | 9.79E−06 | 1.61E−05 | 2.15E−07 | 8.81E−07 | 2.79E−05 | III |
| S8 | 5.90E−07 | 4.82E−08 | 2.15E−05 | 1.66E−05 | 2.21E−07 | 4.14E−07 | 3.94E−05 | III |
| S9 | 6.62E−07 | 5.41E−08 | 8.86E−06 | 1.33E−05 | 1.77E−07 | 3.42E−07 | 2.34E−05 | III |
| S10 | 9.35E−07 | 7.64E−08 | 1.36E−05 | 1.53E−05 | 2.03E−07 | 4.59E−07 | 3.05E−05 | III |
| S11 | 5.97E−07 | 4.88E−08 | 6.81E−06 | 2.00E−05 | 2.66E−07 | 3.41E−07 | 2.81E−05 | III |
| S12 | 1.12E−06 | 9.14E−08 | 1.46E−05 | 1.50E−05 | 1.99E−07 | 6.67E−07 | 3.16E−05 | III |
| S13 | 9.33E−07 | 7.63E−08 | 8.33E−06 | 2.05E−05 | 2.73E−07 | 3.67E−07 | 3.05E−05 | III |
| S14 | 8.83E−07 | 7.21E−08 | 1.09E−05 | 1.51E−05 | 2.01E−07 | 4.34E−07 | 2.76E−05 | III |
| S15 | 1.98E−06 | 1.62E−07 | 1.03E−05 | 1.65E−05 | 2.19E−07 | 6.25E−07 | 2.98E−05 | III |
| S16 | 9.38E−07 | 7.66E−08 | 4.80E−05 | 1.61E−05 | 2.14E−07 | 9.30E−07 | 6.63E−05 | IV |
| S17 | 4.59E−07 | 3.75E−08 | 1.51E−05 | 1.31E−05 | 1.75E−07 | 5.27E−07 | 2.94E−05 | III |
| S18 | 9.17E−07 | 7.50E−08 | 1.13E−05 | 2.07E−05 | 2.75E−07 | 4.99E−07 | 3.37E−05 | III |
| S19 | 1.38E−06 | 1.13E−07 | 2.18E−05 | 2.27E−05 | 3.02E−07 | 6.48E−07 | 4.70E−05 | III |
| S20 | 1.05E−06 | 8.61E−08 | 1.39E−05 | 1.07E−05 | 1.42E−07 | 6.96E−07 | 2.65E−05 | III |

**Table S6.** Reliability degrees of carcinogenic risk caused by trace element in different risk levels.

| Lake | Trace Element | Grade I | Grade II | Grade III | Grade IV |
|---|---|---|---|---|---|
| Dongting Lake | Cd | 0.84 | 0.16 | | |
| | Cr | | | 1 | |
| | As | | | 0.26 | 0.74 |
| | Pb | 1 | | | |
| Pear River Estuary | Cd | 0.37 | 0.63 | | |
| | Cr | | | 1.00 | |
| | Pb | 0.42 | 0.58 | | |

**Table S7.** Differences of carcinogenic risk level in each sampling sites between fuzzy and certainty assessment.

| Lake | Sampling Sites | Reliability Degree Based on Fuzzy Assessment | | | | | | Certainty Assessment |
|---|---|---|---|---|---|---|---|---|
| | | Reliability Degree | | | | | Risk Level | Risk Level |
| | | I | II | III | IV | V | | |
| Dongting Lake | Yugong Temple | | | | 0.28 | 0.72 | V | V |
| | Lujiao | | | | 0.29 | 0.71 | V | V |
| | Nanzui | | | 0.19 | 0.77 | 0.04 | IV | IV |
| | Xiaohezui | | | 0.19 | 0.77 | 0.04 | IV | IV |
| | Hengling Lake | | | 0.03 | 0.61 | 0.36 | IV | IV |
| | Wanzi Lake | | | 0.07 | 0.65 | 0.29 | IV | IV |
| | Jiangjiazui | | | 0.15 | 0.73 | 0.11 | IV | IV |
| | East Dongting Lake | | | | 0.35 | 0.65 | V | V |
| | Bian Mountain | | | | 0.38 | 0.62 | V | V |
| | Yueyang Tower | | | | 0.42 | 0.58 | V | V |
| | Outlet of Dongting Lake | | | | 0.40 | 0.60 | V | V |
| Pear River Estuary | Jiaomen | | | 0.68 | 0.32 | | III | III |
| | Hongqimen | | | 1.00 | | | III | III |
| | Humen1 | | | 1.00 | | | III | III |
| | Humen2 | | | 1.00 | | | III | III |
| | Modaomen | | 0.14 | 0.86 | | | III | III |
| | Yamen | | | 1.00 | | | III | III |
| | Jitimen | | 0.35 | 0.65 | | | III | III |

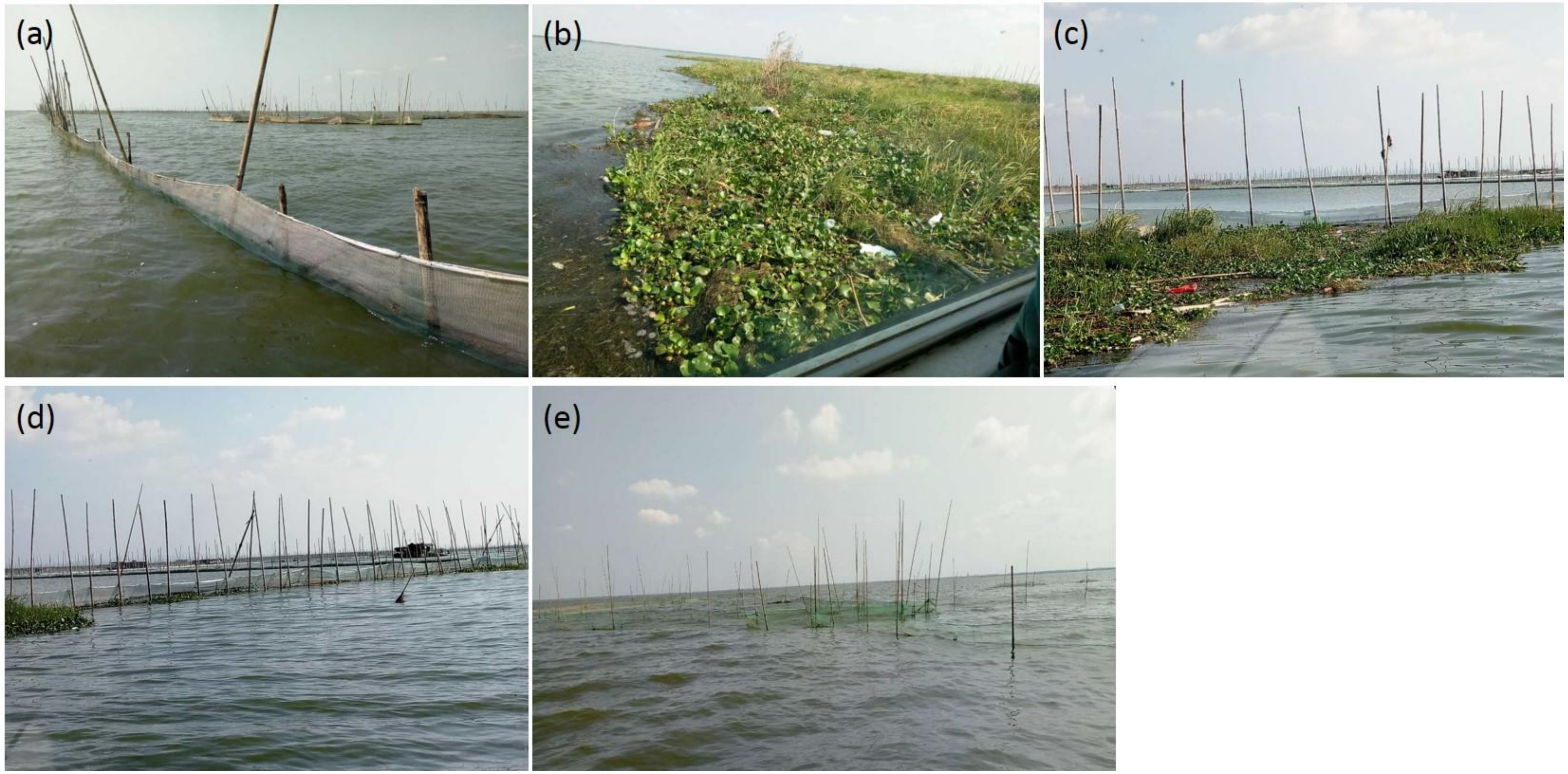


**Figure S1.** Pictures of surrounding conditions in sites S8 (a), S13 (b), S16 (c, d) and S18 (e).
